# Identification of an immune gene signature for predicting the prognosis of patients with uterine corpus endometrial carcinoma

**DOI:** 10.1186/s12935-020-01560-w

**Published:** 2020-11-09

**Authors:** Cankun Zhou, Chaomei Li, Fangli Yan, Yuhua Zheng

**Affiliations:** 1 Department of Gynecology, Southern Medical University Affiliated Maternal & Child Health Hospital of Foshan, Foshan, 528000 Guangdong China; 2 Department of Obstetrics and Gynecology, Southern Medical University Affiliated Maternal & Child Health Hospital of Foshan, Foshan, 528000 Guangdong China; 3 School of Medicine, Southern Medical University, Guangzhou, 510515 China; 4 Department of Gynecology, Shaoxing People’s Hospital, Shaoxing, 312000 Zhejiang China

**Keywords:** Uterine corpus endometrial carcinoma, TCGA, Immune gene, Prognosis

## Abstract

**Background:**

Uterine corpus endometrial carcinoma (UCEC) is a frequent gynecological malignancy with a poor prognosis particularly at an advanced stage. Herein, this study aims to construct prognostic markers of UCEC based on immune-related genes to predict the prognosis of UCEC.

**Methods:**

We analyzed expression data of 575 UCEC patients from The Cancer Genome Atlas database and immune genes from the ImmPort database, which were used for generation and validation of the signature. We constructed a transcription factor regulatory network based on Cistrome databases, and also performed functional enrichment and pathway analyses for the differentially expressed immune genes. Moreover, the prognostic value of 410 immune genes was determined using the Cox regression analysis. We then constructed and verified a prognostic signature. Finally, we performed immune infiltration analysis using TIMER-generating immune cell content.

**Results:**

The immune cell microenvironment as well as the PI3K-Akt, and MARK signaling pathways were involved in UCEC development. The established prognostic signature revealed a ten-gene prognostic signature, comprising of *PDIA3*, *LTA*, *PSMC4*, *TNF*, *SBDS*, *HDGF*, *HTR3E*, *NR3C1*, *PGR*, and *CBLC*. This signature showed a strong prognostic ability in both the training and testing sets and thus can be used as an independent tool to predict the prognosis of UCEC. In addition, levels of B cells and neutrophils were significantly correlated with the patient’s risk score, while the expression of ten genes was associated with immune cell infiltrates.

**Conclusions:**

In summary, the ten-gene prognostic signature may guide the selection of the immunotherapy for UCEC.

## Background

Uterine corpus endometrial carcinoma (UCEC) is one of the most prevalent malignant tumors in women. According to the current global cancer statistics, endometrial cancer has an incidence of about 4.4% [1], with related morbidity and mortality showing an increase annually despite the recent advances in treatment. This has been attributed primarily due to the lack of biomarkers for early diagnosis and prognosis prediction for this condition [2]. Previous studies have elucidated that the Grade, Stage, and TNM staging of UCEC are closely related to disease prognosis. However, some patients may manifest different clinical outcomes within the same stage group, implying that the clinical prognostic information generated through traditional clinical-pathological staging is insufficient. Therefore, in this context, the identification of highly accurate, reliable, and sensitive markers is critical for improving the prognosis of UCEC patients.

Recent studies have demonstrated the important role played by tumor microenvironment (TME)-stromal cells in tumor proliferation, invasion, and metastasis. These cells are closely related to the prognosis of the disease [3, 4]. In addition, host immune responses, with multiple immune cell infiltrations, are one of the main participants in TME [5, 6]. Several studies have hypothesized that UCEC may be associated with long-term inflammatory stimuli, suggesting that the endometrium and menstrual cycles are essentially a chronic inflammatory process involving immune cells [7, 8]. Elsewhere, the effect of immune cell-derived cytokines on the survival outcomes of UCEC patients has been described [9–11]. However, the role of immune-related genes on the systematic prediction of overall survival and response to immunotherapy in UCEC remains enigmatic.

Current and emerging knowledge of tumor molecular biology has led to the development of numerous clinical therapeutic approaches for cancer treatment. Besides, attempts have been made to efficiently and accurately assess the effects of therapies, mainly through preclinical models that simulate characteristics of different types of cancers. For instance, Muhammad et al. [12] demonstrated the anti-proliferative activity of bitter melon extract (BME) in breast cancer cells using homozygous and xenograft mouse models. Furthermore, advances in molecular techniques applied to different available preclinical models have greatly increased our understanding of endometrial cancer biology [13]. Presently, the sequencing of the human genome and DNA microarray development has revealed the identification of candidate genes of prognostic or therapeutic value. For example, the Cancer Genome Atlas (TCGA) database provides comprehensive data on the molecular basis of various types of cancer [14].

In this respect, we aimed to identify prognostic immunomarkers and construct a signature for predicting UCEC. A prognostic risk scoring system was validated by testing set A and B. Specifically, we analyzed RNA-seq data from the TCGA database, as well as immune-related genes downloaded from the Immunology Database and Analysis Portal (ImmPort) databases. Subsequently, we assessed whether these immunity genes were associated with survival outcomes and clinical traits in a subgroup of UCEC patients. Lastly, we explored the relationship between risk scores in UCEC patients with immune cell infiltration, using an abundance of six immune infiltrates from the Tumor Immune Estimation Resource (TIMER) database. Therefore, this study may provide new biomarkers for prognosis and also novel immunotherapy insights for UCEC.

## Materials and methods

### Publicly attainable expression datasets

We downloaded expression data from the TCGA databases. Specifically, RNA-seq and clinical data of 575 UCEC patients were downloaded from the TCGA database (https://portal.gdc.cancer.gov) and used as the training set. Pearson’s correlation coefficient was used to eliminate outliers. Afterward, all patients were randomly assigned to the testing set A (n = 270) and the testing set B (n = 271) based on the complete TCGA data set. Collectively, the 3 sets of data were used to construct the signature.

### Prognosis-related differential expression gene screening

Differential expression genes (DEGs) analysis of the training set was performed using the “limma” package, at a corrected *P* < 0.05 and | logFC | ≥ 1. The resulting data was used to generate heatmaps and volcano plots using R software, version 3.5.3 [15]. Sequence data for immune-related genes and tumor-associated transcription factors were retrieved from the IMMPORT (http://www.immport.org/) and the Cistrome (http://cistrome.org/) [14, 16], which were used for the identification of differentially expressed immune genes and transcription factors (TF), respectively. The resulting datasets were then used to generate heatmaps and volcano plots, as earlier described. Gene Ontology (GO), functional enrichment analyses, and Kyoto Encyclopedia of Genes and Genome (KEGG) pathway analyses were then executed for differential immune genes using “clusterProfiler, org.Hs.e.g.db, plot, ggplot2” packages in R. These analyses were performed at *P *< 0.05 and q < 0.05 as cut-offs.

### Construction of a regulatory network

Here, differentially expressed immunity genes were combined with survival time, while prognosis-related immune genes were evaluated using Cox univariate analysis. We also drew a forest map with a significance filtering standard *P* < 0.01. Thereafter, a correlation was performed with differential TFs at | cor | > 0.4 and *P* < 0.001 as the filtering criteria. Lastly, the resulting data were imported into Cytoscape version 3.7.1 for visualization of the regulatory network.

### Development and validation of the immune prognostic signature for UCEC

The prognostic signature was constructed using the multivariate Cox regression model. Thereafter, the most significant genes concerning prognosis were determined using prognosis-related immune genes. Receiver operating characteristic (ROC) curves for assessing the sensitivity and specificity of the prognostic signature was generated using the “survivalROC” package implemented in R.

Risk scores for each patient were calculated as follows:

$$ Risk\;score = \sum\nolimits_{i = 1}^{n} {exp_{\text{i}} *{\text{coef}}_{\text{i}} } $$

where “n” is the number of prognostic genes, “*exp*_*i*_” is the expression value of the gene i, and “coef_i_” is the gene i coefficient in multivariate Cox regression analysis. Then the median risk value was used to divide the patients into high and low-risk groups, while the Kaplan‐Meier curve was applied to assess the survival difference between the two groups using the log‐rank test. Subsequently, a risk curve was drawn using the “pheatmap” R package.

To determine the feasibility and reliability of the ten-gene prognostic signature, we used testing set A (n = 270) and testing set B (n = 271) of the TCGA sample according to the “Publicly attainable expression datasets” section above. All of the findings are summarized in Additional file 1: Table SA.

### Independent prognostic analysis

Combined with the risk score and clinical data of each sample for independent prognostic analysis, single-factor and multi-factor independent prognostic analysis were used to assess the prognostic value of immune-gene signature and clinical parameters. This was also used to ascertain whether the predictive power of the immune-gene signature was independent of other clinical parameters.

### Relationship among clinical parameters

To assess the association between immunity genes in the prognostic signature and clinical parameters, patients were divided into two subgroups using univariate Cox regression analysis. The first group comprised of patients who were ≤ 55 whereas the second one had those who were > 55 years old. Next, clinically relevant immune genes across patients in the 2 groups were screened and mapped (P < 0.05) using the “beeswarm” package.

### Immunohistochemistry

The Human Protein Atlas (https://www.proteinatlas.org) contains information on tissue and cellular distribution for all 24,000 human proteins. The database applies immunohistochemistry using specific antibodies to analyze differentially expressed proteins in normal and tumor tissues. Herein, we examined this database to analyze profiles of protein expression in ten genes across normal uterine and endometrial carcinoma tissues.

### Correlation between immune cell content and the signature genes

Data on the abundance of six immune infiltrates, including B, CD4+ T, CD8+ T, and dendritic cells as well as neutrophils, and macrophages were retrieved from the TIMER official website (https://cistrome.shinyaoos.io/timer/). Consequently, these data were used to analyze the relationship between risk scores of UCEC patients and the aforementioned immune cells. Furthermore, we explored a correlation between the abundance of immune cells and gene expression as described in [17].

### Statistical analyses

Data on survival analysis was investigated using the Kaplan–Meier curve, with statistical differences determined using the log-rank test. The area under the curve (AUC) of the ROC was used to analyze prediction accuracy of the prognostic signature, whereas effects of clinical traits on overall survival (OS) were assessed using univariate Cox and multivariate Cox regression analyses. The hazard ratio (HR) and 95% confidence interval (CI) were generated using the Cox proportional hazards model. Lastly, Univariate Cox regression analysis was employed to evaluate the correlation between immune cells and gene expression. Data with *P* < 0.05 were considered statistically significant.

## Results

### Identification of DEGs in UCEC

**Fig. 1:**
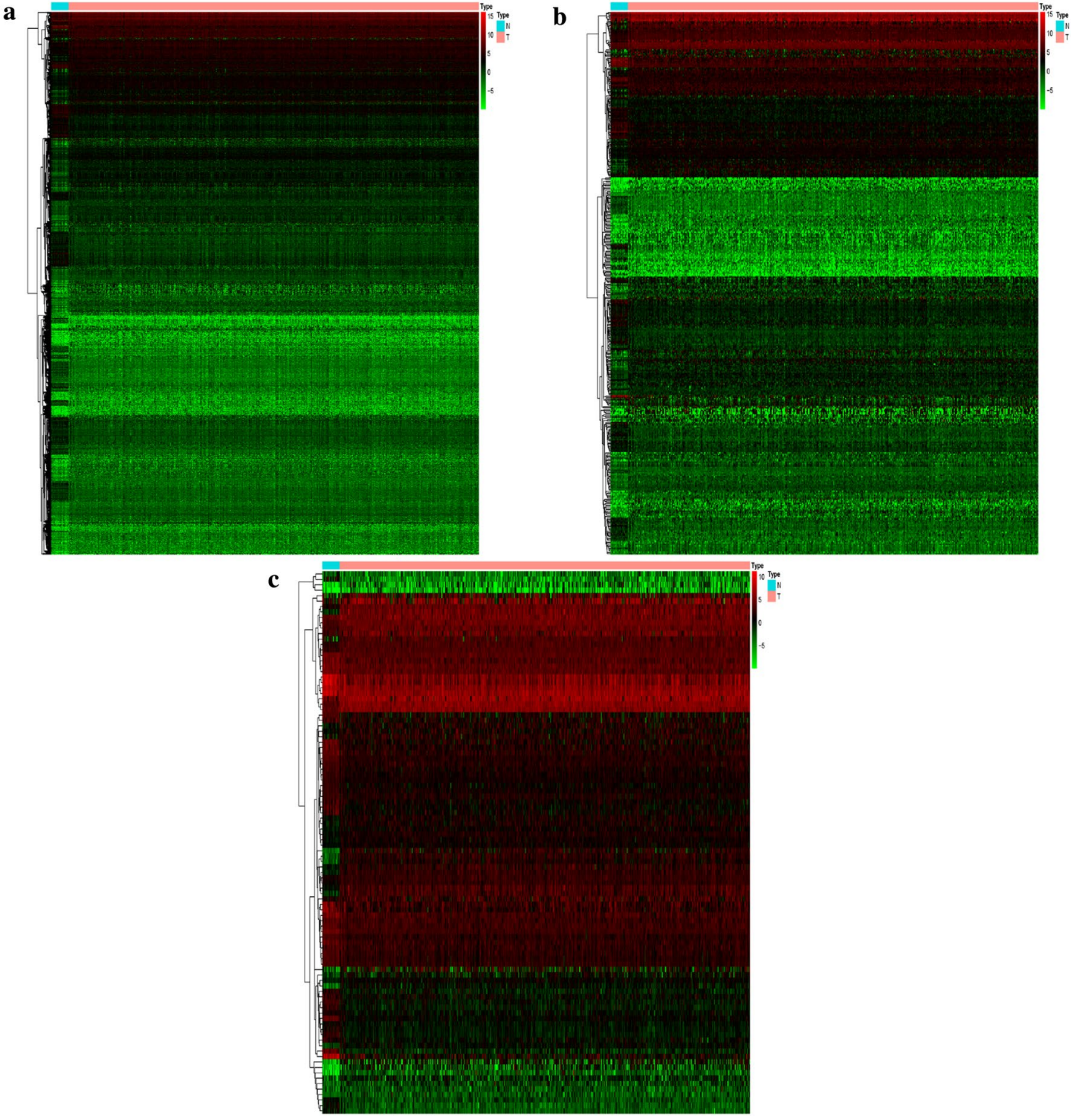
Hierarchical clustering heatmap of DEGs. The genes with higher expression in the heatmap are shown in red, lower expression in green, and genes with the same expression level in black. Tiffany blue represents the adjacent tissue, and the pink represents the cancer tissue. **a** DEGs of RNA-seq gene expression. **b** DEGs of immune genes. **c** DEGs of TFs. DEGs, differentially expressed genes. TFs, transcription factors

**Fig. 2:**
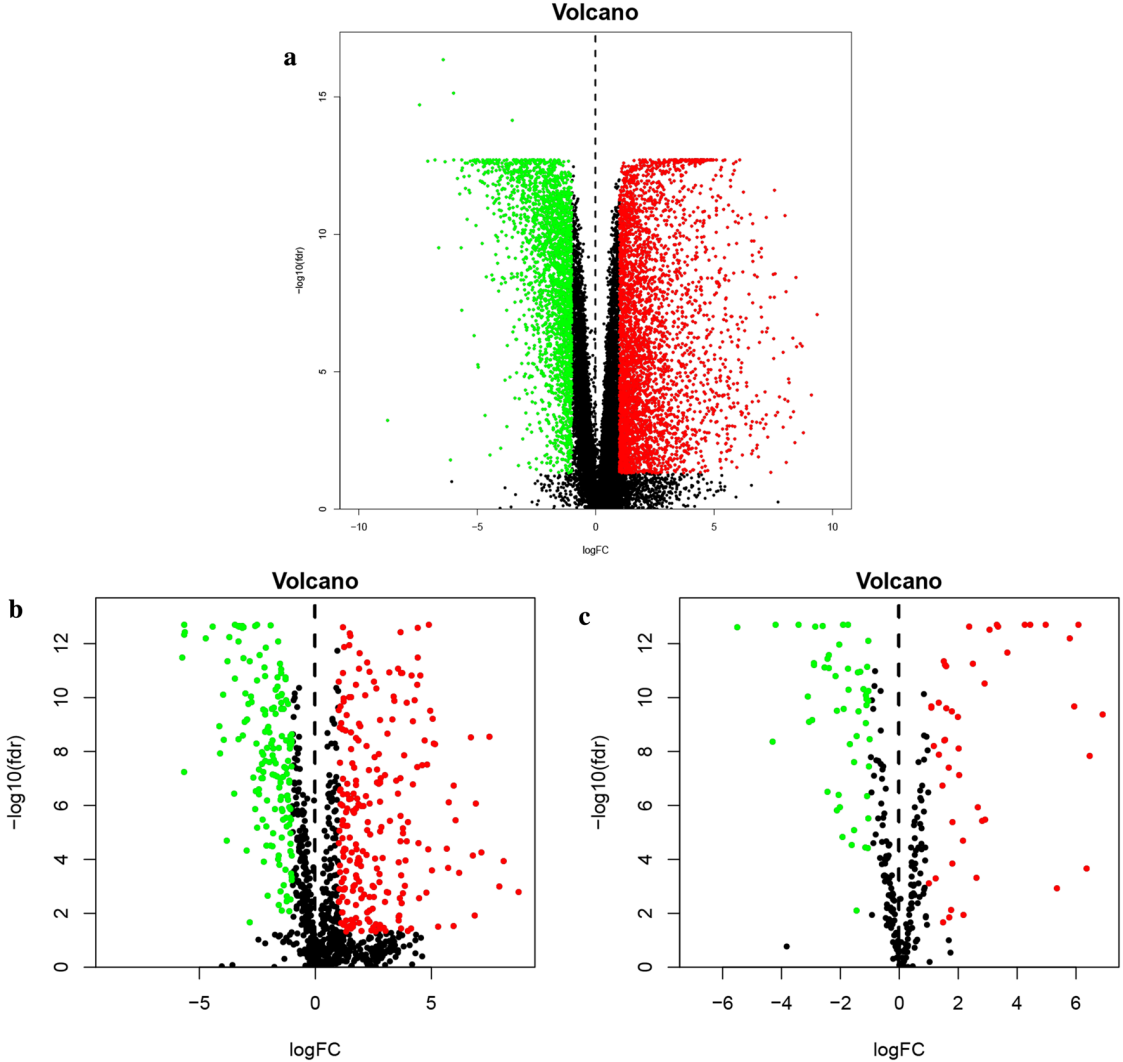
Volcano plot of DEGs. The red dots represent the up-regulated genes screened on the basis of corrected P < 0.05 and logFC ≥ 1. The green dots represent the down-regulated genes screened on the basis of corrected P < 0.05 and logFC ≤ − 1. The black dots represent genes with no significant differences. **a** DEGs of RNA-seq gene expression. **b** DEGs of immune genes. **c** DEGs of TFs. DEGs, differentially expressed genes. TFs, transcription factors. FC, fold change

**Fig. 3:**
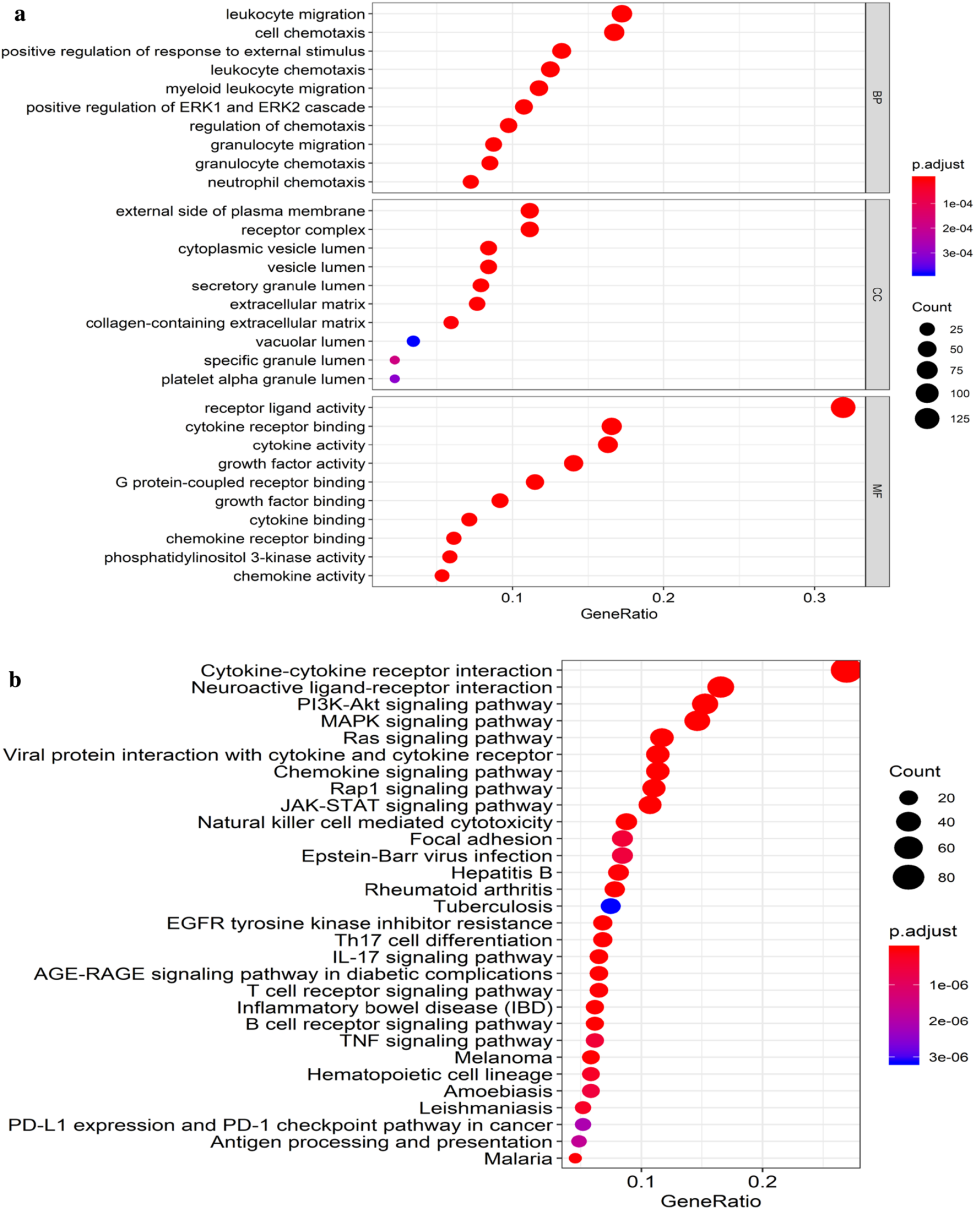
Functional enrichment analysis of DEGs of immune genes. **a** Biological process, Cellular composition, and Molecular function of GO enrichment analysis. **b** Enrichment analysis of KEGG pathway. The results were as follows: BP (Biological process) mainly included the chemotaxis migration of anti-inflammatory cells, including leukocyte and neutrophil, CC (Cellular composition) mainly included extracellular matrix, and MF (Molecular function) mainly included growth factor and cytokine activity. The enriched KEGG pathway mainly included PI3K-Akt, MAPK, Ras, and JAK-STAT signaling pathways. DEG, differentially expressed gene. GO, Gene Ontology. KEGG, Kyoto Encyclopedia of Genes and Genomes

Overall, we identified 6268 DEGs, 410 candidate prognostic immune genes, and 100 differential TFs (Figs. 1 and 2). The differential expression of immune genes in all endometrial cancer samples is summarized in Additional file 1: S1. Enrichment analysis of differentially expressed immunity genes showed that biological processes (BP), mainly chemotaxis migration of anti-inflammatory cells, including leukocyte and neutrophils, were primarily enriched (Fig. 3a). The enriched cellular components (CC) were mainly extracellular matrix whereas the main molecular function (MF) comprised of growth factor and cytokine activity. These findings implied that most differentially expressed immunity genes were associated with UCEC development, progression, and prognosis through immune cells. The enriched top 30 KEGG pathways are given in Fig. 3b. Notably, several signaling pathways involved in UCEC development, including PI3K-Akt, MAPK, Ras, and JAK-STAT, were identified.

### Association between immunity genes and survival rates

**Fig. 4:**
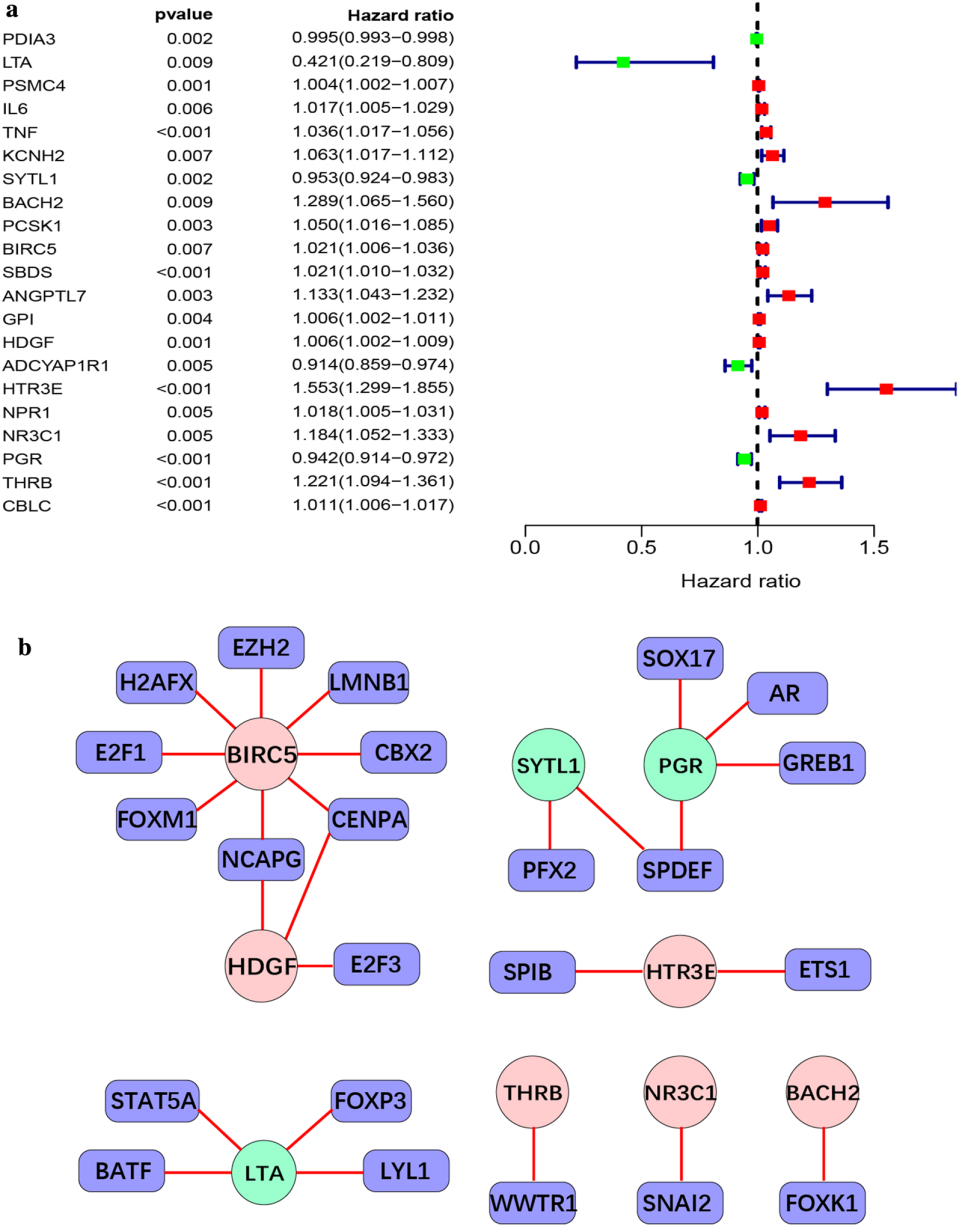
Correlation analysis of prognosis-related immune genes and TFs. **a** Forest of prognosis-related immune genes on the basis of *P* value<0.001. Red means high risk, green means low risk. The higher the Hazard ratio, the higher the prognostic risk. **b** TFs and prognosis-related immune gene regulatory networks on the basis of | cor | > 0.4 and P-value < 0.001. Blue round rectangle represents TFs, dark pink and light green ovals represent high-risk and low-risk prognosis-related immune genes, respectively, and red lines represent positive regulation. TFs, transcription factors

Univariate Cox regression analysis of differentially expressed immunity genes revealed a significant correlation between survival rates and 21 of candidate prognostic immune genes (*P* < 0.01). In particular, the genes with a significant association were *PDIA3*, *LTA*, *PSMC4*, *IL6*, *TNF*, *KCNH2*, *SYTL1*, *BACH2*, *PCSK1*, *BIRC5*, *SBDS*, *ANGPTL7*, *GPI*, *HDGF*, *ADCYAP1R1*, *HTR3E*, *NPR1*, *NR3C1*, *PGR*, *THRB*, and *CBLC.* Among them, *LTA*, *ADCYAP1R1*, *PGR*, *SYTL1*, and *PDIA3* were characterized as low-risk, while the remaining 16 were categorized as high-risk genes. Detailed information of all 21 genes is depicted in Fig. 4a.

Furthermore, to assess the relationship between the 21 prognosis-related immunity genes and TFs, a univariate Cox regression analysis was performed at | cor | > 0.4 and *P* < 0.001, where a TF regulatory network was constructed (Fig. 4b). Notably, the regulatory network diagram that comprised of low- (*PGR*, *SYTL1*, and *LTA*), and high-risk (*BIRC5*, *HDGF*, *HTR3E*, *THRB*, *NR3C1*, *BACH2)* genes, as well as TFs (*AR*, *BATF*, *CBX2*, *CENPA*, *E2F1*, *E2F3*, *ETS1*, *EZH2*, *FOXK1*, *FOXP3*, *GREB1*, *H2AFX*, *LMNB1*, *LYL1*, *NCAPG*, *NR3C1*, *RFX2*, *SNAI2*, *SOX17*, *SPDEF*, *SPIB*, *STAT5A*, and *WWTR1*), elucidated a positive relationship between immunity genes and TFs. Lastly, *BIRC5* was associated with several transcription factors namely *CBX2*, *CENPA*, *E2F1*, *EZH2*, *FOXM1*, *H2AFX*, *LMNB1*, and *NCAPG.*

### The prognostic prediction signature

**Fig. 5:**
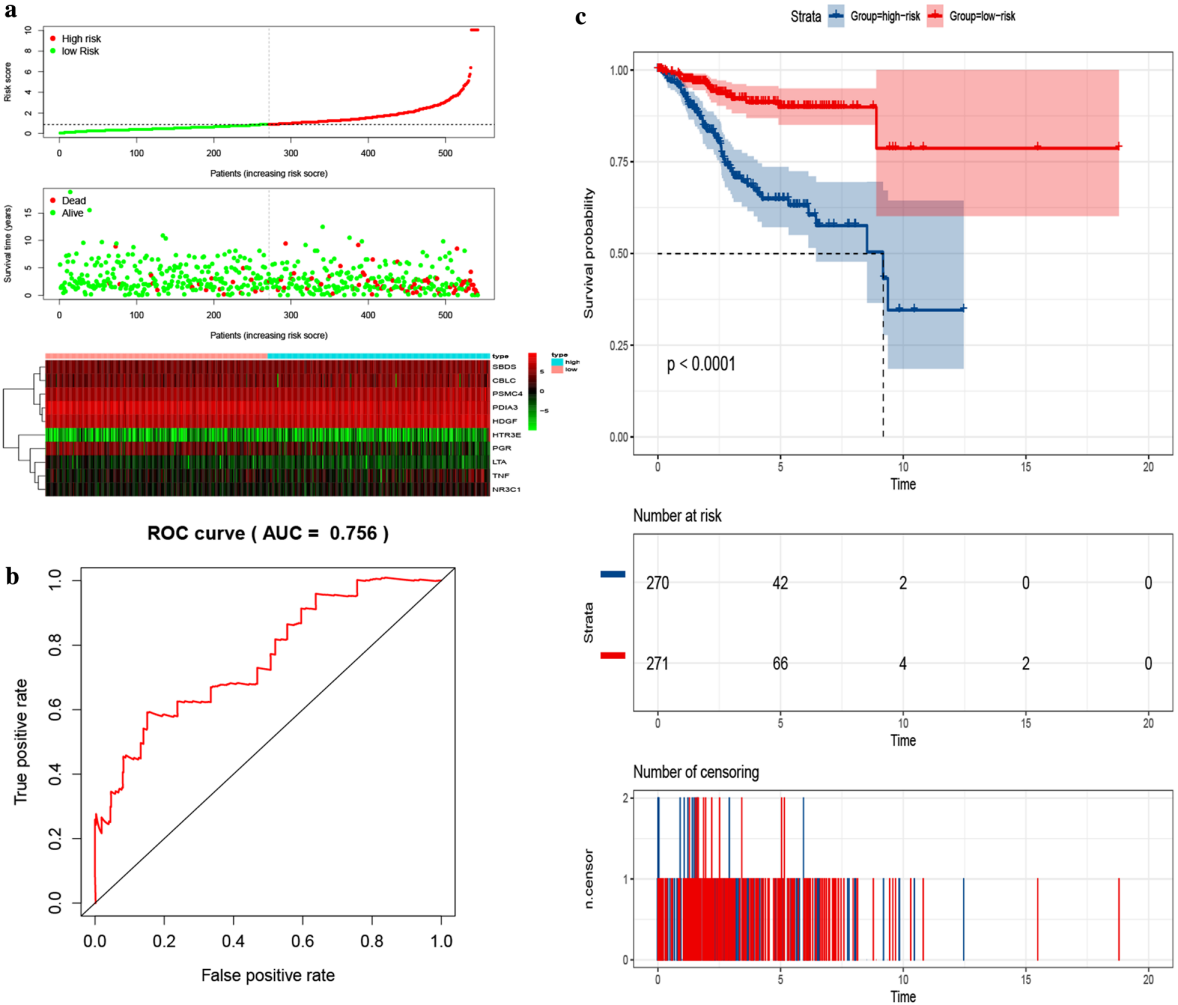
Correlation between the ten-gene prognostic signature and the OS of patients in the training set. **a** The distribution of risk scores, gene expression levels and patient survival status. The black dotted line represents the median cut point and divides patients into low-risk and high-risk groups. **b** ROC curve for judging the accuracy of the signature (AUC = 0.756). **c** Kaplan–Meier curves of OS of high- and low-risk groups (P < 0.0001). OS, overall survival. UCEC, Uterine corpus endometrial carcinoma

To establish a signature for predicting the prognosis of UCEC patients, we employed a Cox regression analysis and identified a ten-gene prognostic signature based on a training set. The genes in the signature included *PDIA3*, *LTA*, *PSMC4*, *TNF*, *SBDS*, *HDGF*, *HTR3E*, *NR3C1*, *PGR*, and *CBLC* (Table 1). We used the prognostic signature to calculate a risk score for each patient, while the median value was used to divide the patients into a high-risk (n = 270) and low-risk groups (n = 271) (Additional file 2: S2 showed the risk score and immune gene expression per patient of the signature in the training set). The prediction power of the ten-gene prognostic signature for patients in training sets is outlined in Fig. 5, while the distribution of risk scores, gene expression levels, and patient survival status are displayed in Fig. 5a. Remarkably, AUC for the training set was 0.756, indicating good accuracy of the prognostic prediction-values across the ten-gene prognostic signature. From the Kaplan‐Meier curve, lower overall survival rates were recorded for patients in the high-risk compared to those in the low-risk group for the training set (*P *< 0.0001) (Fig. 5c). Besides, 5-year OS rates of 63.1 and 89.9%, were recorded for patients in the high- and low-risk groups, respectively, whereas 9‐year OS rates were 34.6 and 78.7% for patients in the high‐ and low‐risk groups, respectively.

**Table 1:** The prognostic model of prognosis-related 10 immune genes

ID	Coef	HR (95% CI)	P-value
PDIA3	− 0.00226	0.997739 (0.994865–1.000621)	0.124063
LTA	− 1.0635	0.345246 (0.170688–0.698318)	0.003086
PSMC4	0.003143	1.003148 (1.000176–1.00613)	0.037905
TNF	0.02748	1.027862 (1.00758–1.048551)	0.006879
SBDS	0.017278	1.017428 (1.00557–1.029426)	0.00387
HDGF	0.002992	1.002996 (0.999056–1.006952)	0.136281
HTR3E	0.548394	1.730472 (1.42863–2.096087)	2.05E−08
NR3C1	0.153559	1.165977 (0.996923–1.363698)	0.054681
PGR	− 0.02721	0.973152 (0.945302–1.001823)	0.066204
CBLC	0.01077	1.010828 (1.004709–1.016984)	0.000508

*Coef* coefficient, *HR* hazard ratio, *CI* confidence interval

### Validation of the ten-gene prognostic signature in UCEC

**Fig. 6:**
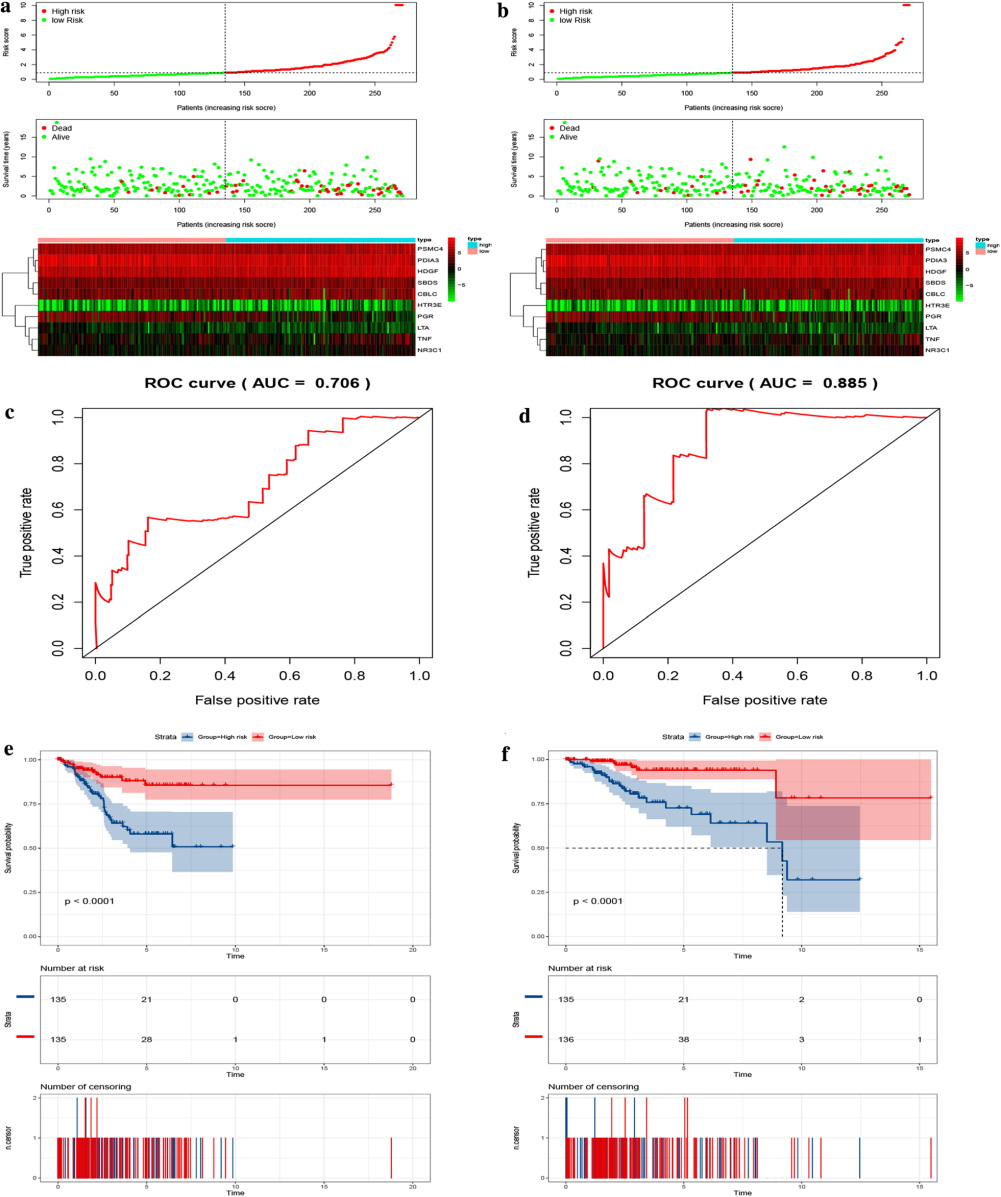
Validation of the ten-gene prognostic signature in the testing set A and testing set B. **a** The distribution of risk scores, gene expression levels and patient survival status in the testing set A. **b** The distribution of risk scores, gene expression levels and patient survival status in the testing set B. **c** ROC curve for judging the accuracy of the signature in the testing set A (AUC = 0.706). **d** ROC curve for judging the accuracy of the signature in the testing set B (AUC = 0.885). **e** Kaplan–Meier curves of OS of high- and low-risk groups in the testing set A (P < 0.0001). **f** Kaplan–Meier curves of OS of high- and low-risk groups in the testing set B (P < 0.0001). OS, overall survival

To determine the feasibility and reliability of the ten-gene prognostic signature, we validated it using testing set A (n = 270) and testing set B (n = 271). In the testing sets A and B, a shorter overall survival rate was noted for patients in the high risk compared to those in the low-risk groups (*P *< 0.0001) (Fig. 6e, f). The AUC for the testing set A and B were 0.706 and 0.885 (Fig. 6c, d), respectively, suggesting that the signature strongly predicts overall survival in UCEC patients (Additional files 3, 4) showed the risk score and immune gene expression per patient of the signature in the testing set A and the testing set B, respectively.)

### The ten-gene prognostic signature is an independent prognostic factor

**Fig. 7:**
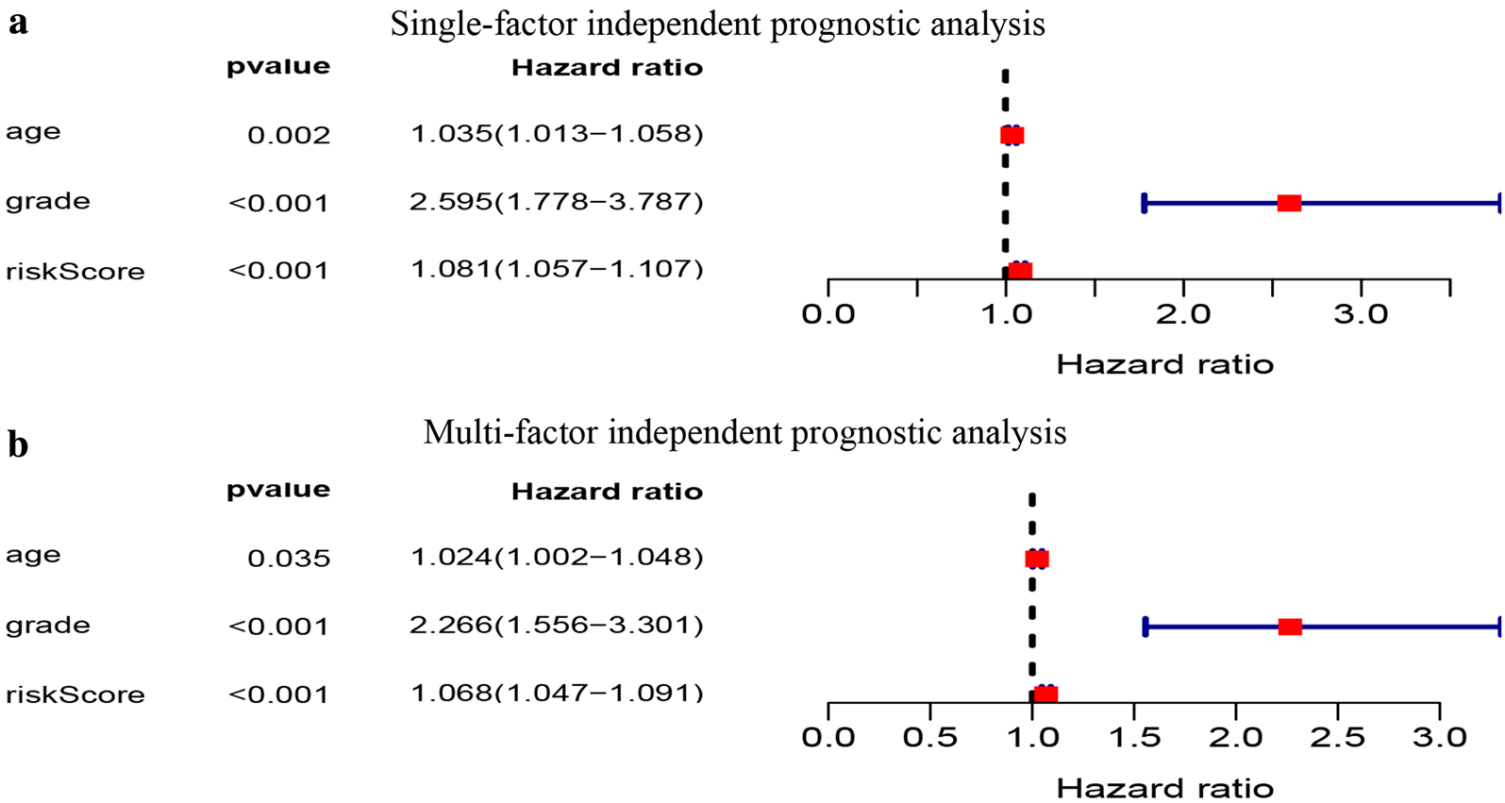
Univariate and multivariate Cox regression analysis of the ten-gene prognostic signature of UCEC patients in TCGA Cohort. **a** Age (P = 0.002), grade (P < 0.001), and the risk score derived from the signature (P < 0.001) were all significantly related to the prognosis by the Univariate Cox regression analysis. **b** Age (P = 0.035), grade (P < 0.001), and the risk score derived from the signature (P < 0.001) were all significantly related to the prognosis by the Multivariate Cox regression analysis. UCEC, Uterine corpus endometrial carcinoma

To determine whether the signature risk score was an independent prognostic factor for patient survival, we employed univariate and multivariate Cox regression analyses. Results demonstrated *P* < 0.05, across both analyses, indicating that the risk score derived from the signature can be independent of other clinical traits, and thus an independent prognostic factor. In addition, univariate Cox regression analysis showed that age (*P* = 0.002, hazard ratio = 1.035) and grade (*P *< 0.001, hazard ratio = 2.595) were significantly associated with prognosis. Of note, the prognosis of patients was worse with an increase in age and grade (Fig. 7).

### Clinical parameters, immunohistochemical examination

**Fig. 8:**
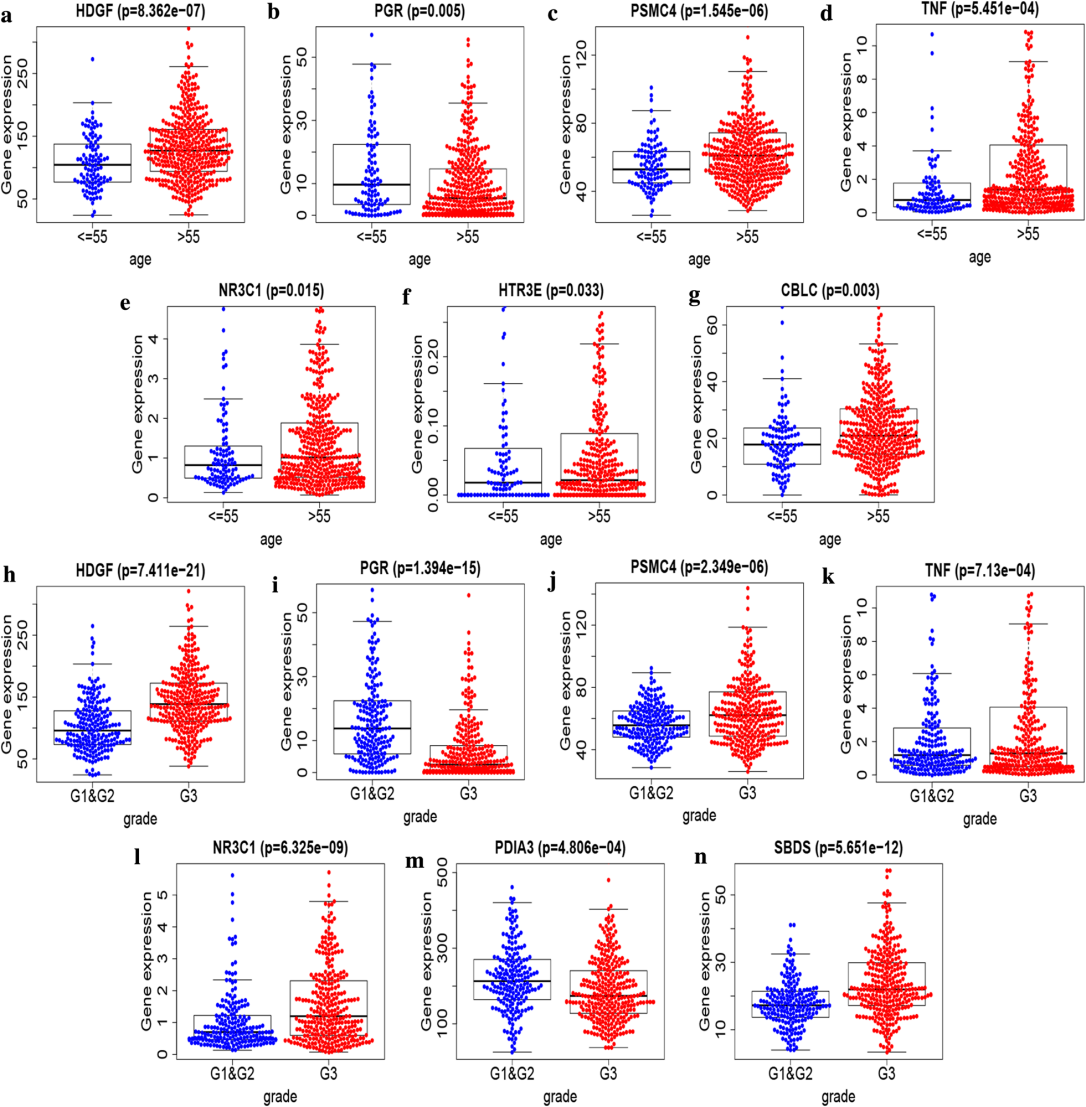
Gene expression levels of **a** HDGF, **b** PGR, **c** PSMC4, **d** TNF, **e** NR3C1, **f** HTR3E, and **g** CBLC between different age of UCEC. The expression of HDGF (P = 5.054e−06), PGR (P = 0.04), PSMC4 (P = 3.257e−04), TNF (P = 5.94e−06), NR3C1 (P = 0.015), HTR3E (P = 0.033) and CBLC (P = 0.003) was significantly correlated with age, while the expression of HDGF, PSMC4, TNF, NR3C1, HTR3E and CBLC increased with age. Gene expression levels of **h** HDGF, **i** PGR, **j** PSMC4, **k** TNF, **l** NR3C1, **m** PDIA3, and **n** SBDS between different clinical grade of UCEC. HDGF (P = 7.411e − 21), PGR (P = 1.394e−15), PSMC4 (P = 2.349e−06), TNF (P = 7.13e−04), NR3C1 (P = 6.325e−09), PDIA3 (P = 4.806e−04), and SBDS (P = 5.651e−12) was significantly associated with grade. Moreover, an increase in grade resulted in upregulation of HDGF, PSMC4, TNF, NR3C1, and SBDS. Abbreviation: UCEC, Uterine corpus endometrial carcinoma

**Fig. 9:**
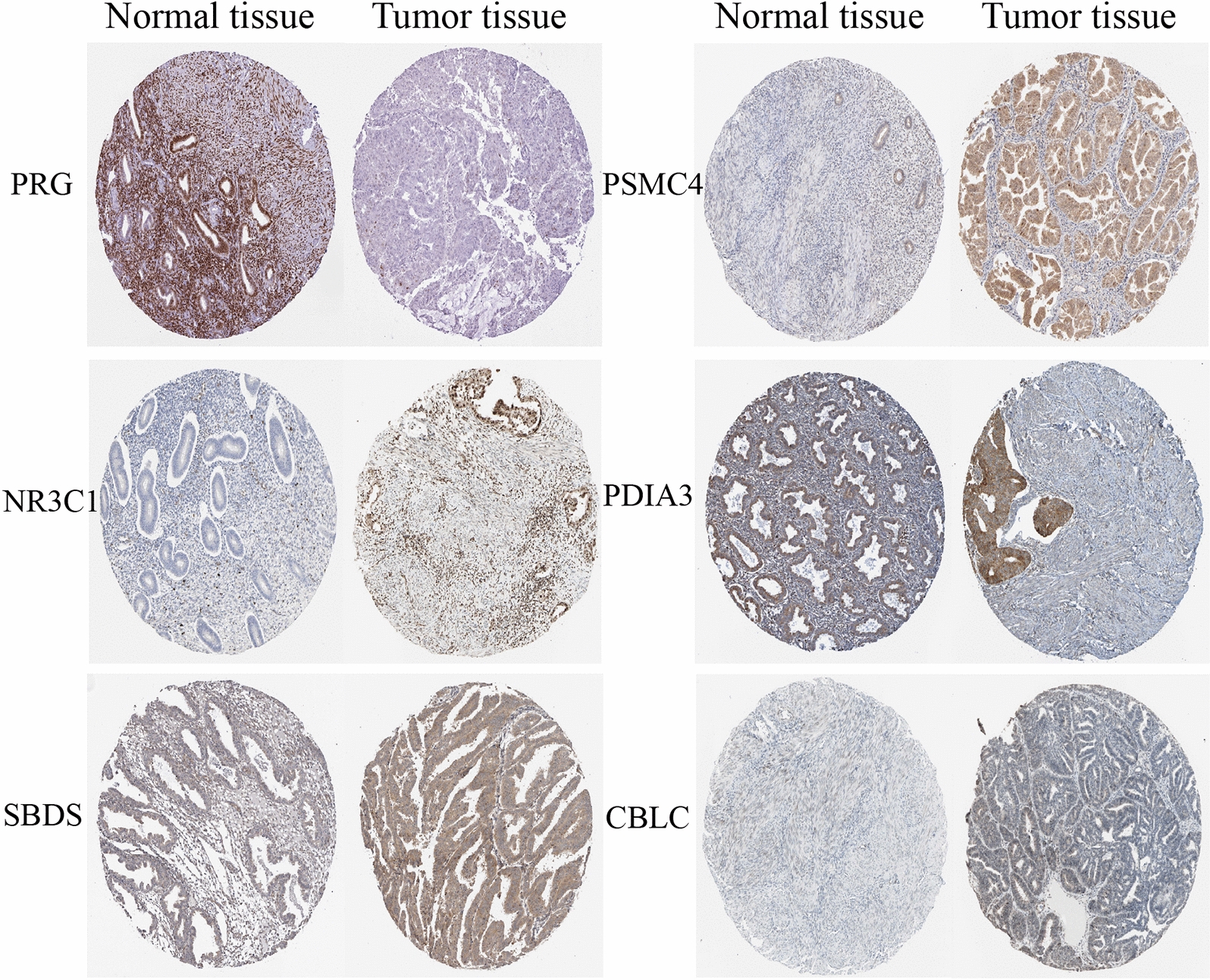
The level of ten genes in endometrial cancer patients in protein level (The Human Protein Atlas). Immunohistochemical examination for expression of PSMC4, NR3C1, SBDS, and CBLC were significant up-regulated in endometrial cancer tissue compared with normal tissues, while PGR and PDIA3 expression were significantly down-regulated in endometrial cancer tissues compared to normal tissues

The correlation between immune genes involved in the signature and clinical traits was assessed using Univariate Cox regression analysis. Here, patients were divided into two groups, based on clinical traits: Group 1 (comprised of patients aged < 55 and ≥ 55) and Group 2 (G1 & G2 and G3). Results revealed a significant correlation between HDGF (*P* < 0.001), PGR (*P* = 0.04), PSMC4 (*P* < 0.001), TNF (*P* < 0.001), NR3C1 (*P* = 0.015), HTR3E (*P* = 0.033) and CBLC (*P* = 0.003) with age, whereas expression of HDGF, PSMC4, TNF, NR3C1, HTR3E, and CBLC increased with age. On the other hand, HDGF (*P* < 0.001), PGR (*P* < 0.001), PSMC4 (*P* < 0.001), TNF (*P* < 0.001), NR3C1 (*P* < 0.001), PDIA3 (*P* < 0.001), and SBDS (*P* < 0.001) were significantly associated with grade. Moreover, an increase in grade resulted in the upregulation of HDGF, PSMC4, TNF, NR3C1, and SBDS (Fig. 8). Immunohistochemical analysis based on The Human Protein Atlas database enumerated a significant upregulation of PSMC4, NR3C1, SBDS, and CBLC in endometrial cancer tissues, relative to normal tissues. On the other hand, immunohistochemical analysis of PGR and PDIA3 expression showed significant downregulation of these factors in endometrial cancer compared to normal tissues (Fig. 9).

### Immune infiltrates analysis of the signature genes in patients with UCEC

**Fig. 10:**
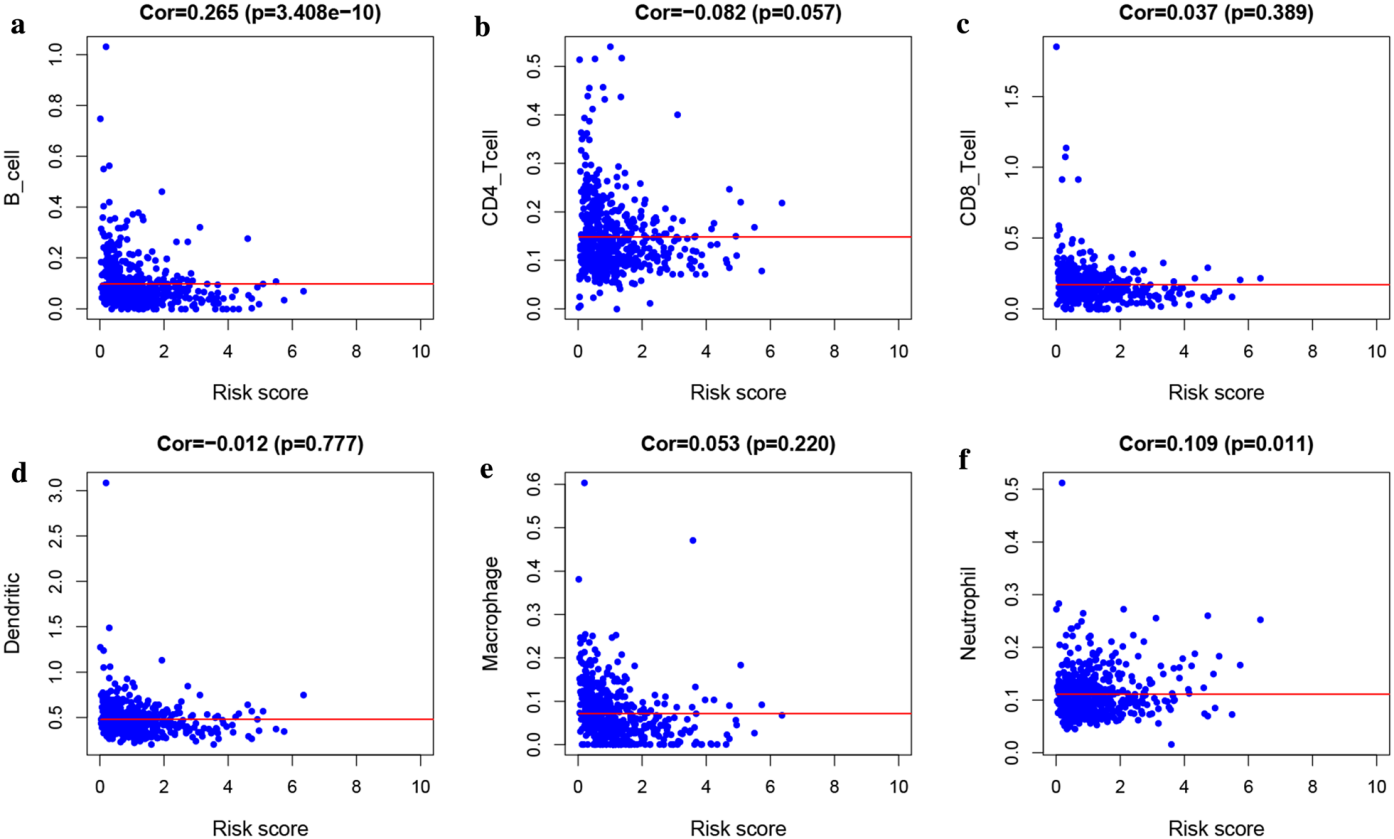
Association between the risk score of the ten-gene prognostic signature and the abundance of 6 immune infiltrates, where B cells (**a**) and Neutrophils (**f**) were significantly correlated with the patient’s risk score and were positively correlated, while CD4_Tcell (**b**), CD8_Tcell (**c**) Dendritic (**d**), and Macrophage (**e**) were not significantly correlated with the patient’s risk score

**Fig. 11:**
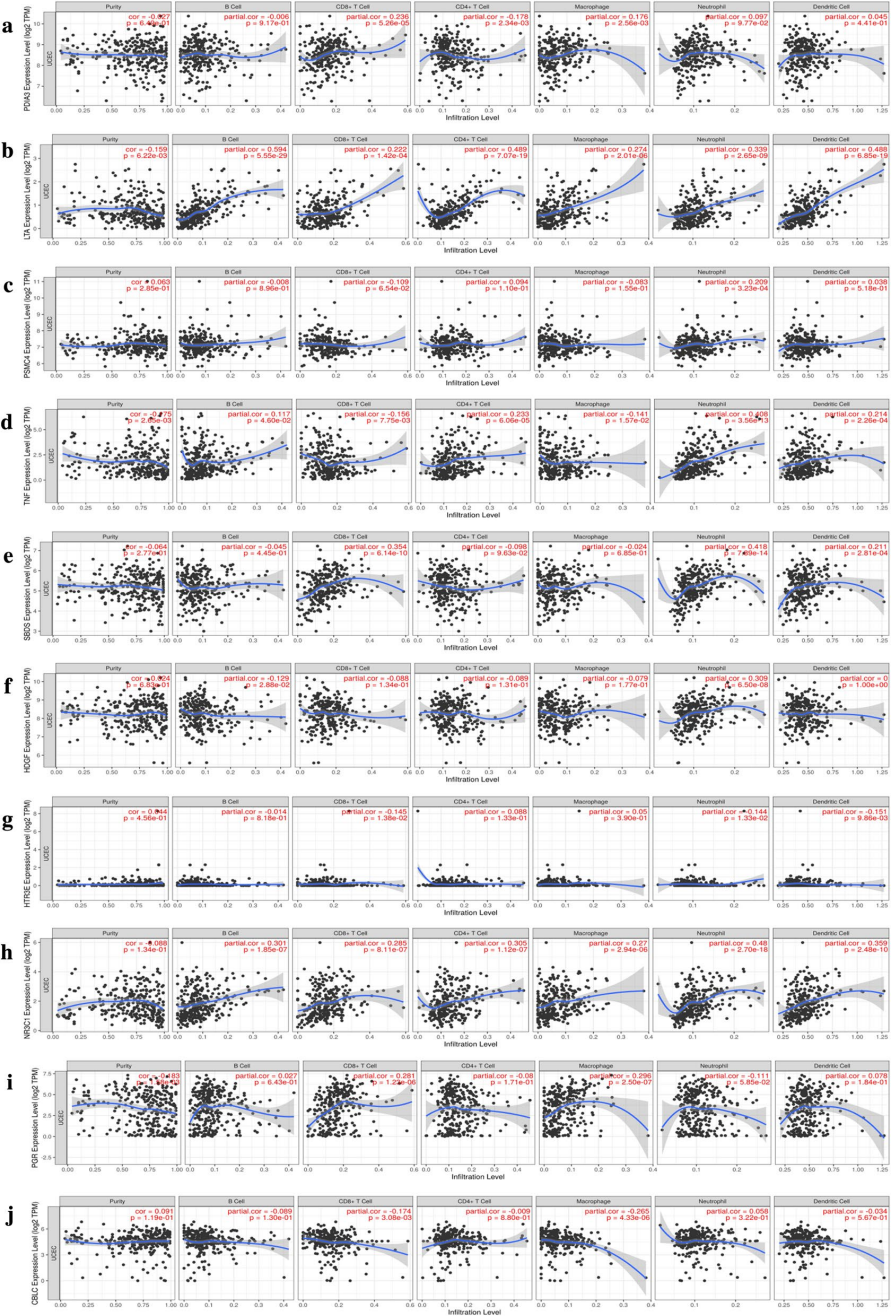
The correlation between **a** PDIA3, **b** LTA, **c** PSMC4, **d** TNF, **e** SBDS, **f** HDGF, **g** HTR3E, **h** NR3C1, **i** PGR, **j** CBLC and the immune infiltration level in UCEC. Abbreviation: UCEC, Uterine corpus endometrial carcinoma

Herein, a correlation analysis between risk scores in UCEC patients with abundance of six immune infiltrations indicated a significant positive association between B cells (*P* = 3.408e−10, cor = 0.265) and neutrophils (*P* = 0.011, Cor = 0.109) with the patient’s risk score (Fig. 10). To explain this relationship, we analyzed infiltration abundance, and noted a positive relationship between B cells and expression of LTA (Cor = 0.594, *P* = 5.55e−29) (Fig. 11b), TNF (Cor = 0.117, *P* = 4.60e−02) (Fig. 11d), and NR3C1 (Cor = 0.301, P = 1.85e−07) (Fig. 11h). Moreover, the infiltration abundance of neutrophils was positively correlated with expression of LTA (Cor = 0.339, *P* = 2.65e−09) (Fig. 11b), PSMC4 (Cor = 0.209, *P* = 3.23e−04) (Fig. 11c), TNF (Cor = 0.408, *P *= 3.56e−13) (Fig. 11d), SBDS (Cor = 0.418, *P *= 7.89e−14) (Fig. 11e), HDGF (Cor = 0.309, *P *= 6.50e−08) (Fig. 11f), and NR3C1 (Cor = 0.48 *P* = 2.70e-18) (Fig. 11h).

## Discussion

Numerous reports have described the relationship between differentially expressed genes and various aspects of tumors, including tumorigenesis and prognosis [18–20]. However, a vast majority of genes implicated in playing a central role in predicting tumor prognosis are limited by certain factors, such as insufficient sample sizes. In this study, we employed a large sample size comprising of TCGA genome-wide expression data to develop a ten-gene prognostic signature for UCEC patients. The signature is anticipated to guide the identification of potential biomarkers that can monitor the prognosis and response to immunotherapy in UCEC patients.

Our results revealed an association between differentially expressed immune genes with immune cell responses to extracellular matrix and tumor microenvironment in UCEC, which was parallel with previous studies [21]. In addition, KEGG enrichment analysis showed that UCEC may be associated with several well-known cancer-related pathways, including the PI3K-Akt, MAPK, Ras, and JAK-STAT signaling pathways. Previous studies have demonstrated the activation of the PI3K-AKT signaling pathway in UCEC patients, as well as its role in promoting tumor development [22]. Also, several studies have revealed multiple factors that activate MAPK, Ras, and JAK-STAT signaling pathways, thereby mediating proliferation, infiltration, and other biological behaviors to promote the occurrence and progression of UCEC [23–25]. Our TF-related regulatory network showed that BIRC5 was positively regulated by multiple TFs, and BIRC5 was a high-risk gene. This was consistent with previous studies demonstrating that the up-regulation of BIRC5 leads to the development and progression of many malignant tumors in humans [26]. Elsewhere, BIRC5 was noted to be overexpressed in more than 90% of UCEC [27], while another study demonstrated frequent overexpression of BIRC5 in recurrent UCEC relative to primary tumors [28].

Herein, we developed a ten-gene prognostic signature, comprising of *PDIA3*, *LTA*, *PSMC4*, *TNF*, *SBDS*, *HDGF*, *HTR3E*, *NR3C1*, *PGR,* and *CBLC*, for prediction of overall survival rates in UCEC patients. Our findings indicated that the signature effectively predicted the OS of UCEC patients, with a statistically significant correlation between the training and testing sets. These findings signify the potential of this signature as a powerful prognostic tool for the entire cohort of UCEC patients. In addition to hepatoma-derived growth factor (HDGF) and Protein disulfide-isomerase A3 (PDIA3), the remaining 8 genes have not been well validated in gynecologic oncology, especially in UCEC. The HDGF is a heparin-binding growth factor that has been purported to play a crucial role in the differentiation, growth, and division of various tissues. Several studies have demonstrated its involvement in the occurrence and development of malignant tumors, promoting proliferation and differentiation of tumor cells, as well as enhancing the metastatic ability of tumor cells through EMT [29, 30]. Besides, HDGF was noted to be an independent risk factor for the prognosis of various malignancies such as liver, gastric, cholangiocarcinoma, and non-small cell lung cancers [31]. However, in endometrial cancer, HDGF has been implicated in multiple abnormalities. For example, a higher FIGO stage mediated HDGF upregulation, a potential adverse factor for the progression and prognosis of UCEC [32].

On the other hand, PDIA3, also known as ERp57/GRP58, has been associated with malignant transformation of cells through STAT3 and Wnt signaling pathways. Also, this factor has been closely associated with the occurrence and development of various tumors [33]. Interestingly, PDIA3 has been reported to enhance the ability of cervical and ovarian cancer cells to proliferate and invade, indicating its potential as a sensitive marker for reflecting tumor prognosis during gynecologic oncology [34, 35]. In this work, these two immune genes were key DEGs (*P *< 0.0001), suggesting their possible role in the development and progression of UCEC. Notably, the overall survival rate of patients in the high-risk group was lower than those in the low-risk group, whereas the AUC values showed that a combination of the ten immune genes exhibited a prognostic value in UCEC patients. Of note, our signature was an independent prognostic factor, because the predicted survival rates were not related to other clinical traits.

In the present study, our results revealed that age and grade were associated with the OS of UCEC patients in high-risk factors. This corresponded with previous studies showing that age, stage, and body weight are clinical prognostic factors for UCEC [36]. Conversely, age and grade were also associated with the prognosis of endometrial cancer and were also a high-risk factor for the disease. This finding agrees with the reports of previous studies that have described age, stage, and body weight as clinical prognostic factors for endometrial cancer. Further correlation analysis revealed a significant positive correlation between HDGF and PSMC4 with age and grade. This may be attributed to the fact that the up-regulation of these genes could promote tumor development [37, 38]. In terms of survival prediction, the current staging system is far from accurate at the individual level. Elsewhere, age is not a survival indicator for cancer because older people are less likely to receive adjuvant therapy [39]. Therefore, risk scores present a more reliable tool for the prognosis of UCEC patients compared to age and stage.

Currently, numerous studies have hypothesized the involvement of immune cells and related inflammatory factors in the UCEC interstitial, which is an important component of the tumor inflammatory microenvironment and generates a marked influence on the biological behavior of UCEC [40]. Consequently, we analyzed the relationship between UCEC risk-score and immune cells using immune cell infiltration abundance data from TIMER. Results indicated a significant positive correlation between B cells and neutrophils with the patient’s risk-scores. Furthermore, we found a close relationship between prognostic signature genes and immune cells. Among them, neutrophils were positively correlated with the expression of several genes, including *LTA*, *PSMC4*, *TNF*, *SBDS*, *HDGF*, and *NR3C1*. This phenomenon may be attributed to the secretion of HDGF, which has been shown to promote neutrophil infiltration and induce inflammatory signals [41]. In another study by Wikberg et al. [42] demonstrated that neutrophils of the innate immune system play a significant role in acute inflammation as well as in anti-tumor immune responses. Despite the close association between neutrophil infiltration with other immune cell infiltration, studies have enumerated that neutrophil infiltration may have additional prognostic values in various cancers. For example, neutrophils persist in tissues, during chronic inflammation, causing cancer progression. Several studies have also shown that elevated numbers of neutrophils in many human cancers or a higher neutrophil/lymphocyte ratio (NLR) are associated with poor prognosis possibly because neutrophils secrete matrix metalloproteinase-9 to stimulate the angiogenic activity of cancer cells [43, 44]. Different proportions of infiltrating B cells were included in solid human tumors. Although the search for immune-related factors associated with a cancer diagnosis, prognosis, and survival has been largely limited to T cell responses, recent reports have suggested that B cells may also play critical roles in the prognosis of cancer patients. For example, findings by Schimdit et al. [45, 46] outlined that the B cell marker was the strongest prognostic factor in breast cancer and other human tumors, given the immunoglobulin kappa chain (IGKC) secreted by plasma cells. On the other hand, another work by Nielsen et al. [47] found that increase in CD20 + B cells resulted in higher survival rates of patients with advanced ovarian cancer. Hence, an increase in the risk-score is likely to elevate levels of these two immune cells and thereby influence immune escape or suppression.

Despite the important clinical value of these findings in UCEC, there were several limitations to our study. Firstly, age and grade were the only clinical traits in the TCGA database of UCEC, although related aspects such as stage and TMN may strengthen the value of the identified genes. Secondly, most of the ten-gene prognostic signature and immune cells have rarely been reported in UCEC patients. In this regard, more prospective studies are needed to validate the intrinsic relevance of these genes in the prognosis of UCEC patients.

## Conclusion

In summary, this study aimed to construct immune gene-related prognostic signature and potential functions of immune genes in the signature. Here, we present a ten-gene prognostic signature that is an independent prognostic factor and might complement clinical features and facilitate personalized immunotherapy in UCEC patients.

## Supplementary information

**Additional file 1:** The differential expression of immune genes in all endometrial cancer samples is summarized.

**Additional file 2:** The risk score and immune gene expression per patient of the signature in the training set.

**Additional file 3:** The risk score and immune gene expression per patient of the signature in the testing set A.

**Additional file 4:** The risk score and immune gene expression per patient of the signature in the testing set B.

## Data Availability

The public datasets used in our work can be found on https://portal.gdc.cancer.gov, http://www.immport.org, http://cistrome.org/, https://www.proteinatlas.org, and https://cistrome.shinyaoos.io/timer/.

## Abbreviations

UCEC: Uterine corpus endometrial carcinoma
TME: Tumor microenvironment
BME: Bitter melon extract
TCGA: The Cancer Genome Atlas
ImmPort: Immunology Database and Analysis Portal
TIMER: Tumor Immune Estimation Resource
DEG: Differential expression genes
TF: Transcription factor
GO: Gene Ontology
KEGG: Kyoto Encyclopedia of Genes and Genome
ROC: Receiver operating characteristic
AUC: Area under curve
OS: Overall survival
HR: Hazard ratio
CI: Confidence interval
BP: Biological processes
CC: Cellular components
MF: Molecular function
HDGF: Hepatoma-derived growth factor
PDIA3: Protein disulfide-isomerase A3
NLR: Neutrophil/lymphocyte ratio
IGKC: Immunoglobulin kappa chain
